# Effect of an educational intervention on knowledge and attitude regarding pharmacovigilance and consumer pharmacovigilance among community pharmacists in Lalitpur district, Nepal

**DOI:** 10.1186/s13104-016-2343-5

**Published:** 2017-01-03

**Authors:** Nisha Jha, Devendra Singh Rathore, Pathiyil Ravi Shankar, Shital Bhandary, Rabi Bushan Pandit, Sudesh Gyawali, Mohamed Alshakka

**Affiliations:** 1 Department of Clinical Pharmacology and Therapeutics, KIST Medical College, Lalitpur, Nepal; 2 Department of Pharmacy, NIMS University, Jaipur, Rajasthan India; 3 Department of Pharmacology, Xavier University of School of Medicine, Oranjestad, Aruba; 4 Department of Community Health Sciences, School of Medicine, Patan Academy of Health Sciences, Kathmandu, Nepal; 5 KIST Medical College, Lalitpur, Nepal; 6 Department of Pharmacology, Manipal College of Medical Sciences, Pokhara, Nepal; 7 Department of Pharmacy, Aden University, Aden, Yemen

**Keywords:** Adverse drug reaction, Community pharmacists, Nepal, Pharmacovigilance, Reporting systems

## Abstract

**Background:**

Pharmacovigilance activities are in a developing stage in Nepal. ADR reporting is mainly confined to healthcare professionals working in institutions recognized as regional pharmacovigilance centers. Community pharmacists could play an important role in pharmacovigilance. This study was conducted among community pharmacists in Lalitpur district to examine their knowledge and attitude about pharmacovigilance before and after an educational intervention.

**Methods:**

Knowledge and attitude was studied before, immediately after and 6 weeks following the intervention among 75 community pharmacists. Responses were analysed using descriptive and inferential statistics. A pretested questionnaire having twelve and nine statements for assessing knowledge and attitude were used. The overall scores were obtained by adding the ‘knowledge’ and ‘attitude’ scores and ‘overall’ scores were summarized using median and interquartile range. Wilcoxon signed-rank test for repeated samples was used to compare the differences between knowledge and attitude of the pharmacists before and after the educational program.

**Results:**

Knowledge scores [median (interquartile range)] improved significantly between pre-test [39 (44–46)], post-test [44 (44–44)] and retention period of 6 weeks after the intervention [46 (43–46)]. Knowledge score improved immediately post-intervention among both males [44 (41–47)] and females [44 (43–45)] but the retention scores (after 6 weeks) were higher [46 (42–48)] among males. Attitude scores improved significantly among females [46 (44–48)]. The overall scores were higher among pharmacists from rural areas.

**Conclusion:**

Knowledge and attitude scores improved after the educational intervention. Further studies in other regions of the country are required. The national pharmacovigilance center should promote awareness about ADR reporting among community pharmacists.

**Electronic supplementary material:**

The online version of this article (doi:10.1186/s13104-016-2343-5) contains supplementary material, which is available to authorized users.

## Background

Adverse drug reactions (ADRs) can be a serious threat to the health of the Nepalese population as a variety of medicines like, allopathic, traditional, homeopathic and ayurvedic are available in ever increasing amounts. Strengthening the ADR reporting system, and making ADR reporting by health professionals mandatory can be useful but unfortunately reporting is not mandatory according to the laws and regulations of Nepal [1]. Voluntary reporting of ADRs by healthcare professionals (HCPs) is limited [2]. There is no involvement of community pharmacists (CPs) in the existing ADR reporting system [3]. In the Pokhara valley in western Nepal, a pilot program was conducted to train selected CPs in ADR reporting and pharmacovigilance [4]. During the study period 71 ADRs were reported by the CPs. However, there has been no further development of ADR reporting by CPs.

Nepal is situated between India and China and is divided into mountainous region, hilly region and plain terrain. The difficult terrain causes problems for the population in accessing healthcare facilities. The total population of Nepal in 2011 was 26.5 million [3]. The GDP per capita was US$ 562. Population below 15 years was 37% and above 60 years was 6% of the total population [5]. Urban population was 28.1% of the total population [6]. Adult literacy rate was 65.9% [7].

Nepal produces a significant number of doctors and other health personnel but only a low number stay back as they get better opportunities in the developed world. This brain drain has resulted in a very poor doctor population, pharmacist population and nurse population ratio. There are 10,197 (3.64/10,000 population) medical and dental doctors, 32,846 (11.71/10,000 population) nursing and midwifery personnel and 731 (0.261/10,000 population) licensed pharmacists in Nepal [8]. This low ratio of HCP to patients can result in increased workload creating difficulty in sparing time for ADR reporting. This could be an important reason for underreporting by HCPs in Nepal. Underreporting remains a big problem worldwide among HCPs [9–11]. The pharmacist population ratio in Nepal is 0.3/10,000 while the community pharmacist population ratio is only 0.013/10,000 [8].

### Pharmacy practice in the community

Doctors do not practice in many remote and rural areas of Nepal. The population depends on local practitioners and community pharmacies for healthcare and a large number of community pharmacies have been established. The number of pharmacies is greater than the number of health centres [12, 13]. Community pharmacists do not charge consultation fees unlike medical doctors. This may be a factor responsible for these pharmacies being the first point of contact with the healthcare system for the general public. These factors support and encourage self-medication in the country [13, 14] which may increase the incidence of ADRs.

The number of qualified pharmacists in community pharmacies, capable of delivering quality pharmaceutical services is inadequate [15]. As per the Drug Act of 1978, pharmacists, assistant pharmacists and pharmacy professionals are eligible to work in a community pharmacy after registering with the national drug regulatory authority, the Department of Drug Administration (DDA) [16].

Pharmacists working in community pharmacies have completed either a three year Diploma in Pharmacy (DPharm) after ten years of schooling (assistant pharmacists), or four year Bachelor in Pharmacy (BPharm) degree after twelve years of schooling (pharmacists). However, some have only completed a training and orientation program of 2 weeks, conducted by DDA (pharmacy professionals) [17, 18]. Only pharmacists and assistant pharmacists are eligible for registering with the Pharmacy Council of Nepal [19].

In Nepal with a high prevalence of self-medication and non-doctor prescribing, it is important to expand the existing pharmacovigilance system to involve community pharmacists [1, 14, 20]. A community-based ADR reporting system can play an active role in collecting reports of ADRs occurring in the community [3, 4].

### Pharmacovigilance activities in Nepal

Nepal was recognized as a member of the International Pharmacovigilance Programme in 2007. The national pharmacovigilance centre run by the DDA, coordinates with seven regional pharmacovigilance centres to collect ADR reports which are forwarded to the Uppsala Monitoring Centre in Sweden, a centre for international service and scientific research towards patient safety [21]. Till date, the ADR reporting system does not involve community pharmacists and consumers and depends on voluntary reporting from other healthcare professionals, predominantly doctors, hospital pharmacists and nurses [22].

### Rationale of the study

Factors like knowledge about medicines, cost of medicines, regulatory systems, laws, cultural issues and community beliefs can influence medicine use by consumers. They can also be influenced by the commercial promotion of medicines and communication with the prescriber and dispenser [20]. Nepal has many medicine use problems, for example: polypharmacy, use of expired drugs, irrational combination drugs, and overuse of antibiotics, vitamins/herbal remedies and prescribing using brand names [13, 14]. These combined with lack of information to patients about the proper handling and use of drugs can lead to serious consequences like ADRs and interactions [14]. Studies show that community pharmacists may have an important role in pharmacovigilance [3, 23]. To participate effectively however, community pharmacists should have adequate knowledge and proper attitudes towards pharmacovigilance. The present study has the objective of evaluating knowledge and attitude of community pharmacists regarding pharmacovigilance and consumer pharmacovigilance and compares the scores among different subgroups before, immediately after and six weeks following an educational intervention.

## Methods

### Study site and study period

The study was conducted from August 2014 to June 2015 among community pharmacists in Lalitpur district, one of the seventy-five districts of Nepal. The district, with Patan as its headquarters, covers an area of 385 km^2^ and is one of the three districts in the Kathmandu Valley, along with Kathmandu and Bhaktapur. Its population was 466,784 in the initial 2011 census tabulation [7]. Lalitpur District has two medical colleges, various other colleges and other institutions of higher learning [7].

### Study design

The design was cross sectional conducted at three points in time, before, immediately after and 6 weeks following an educational intervention and the study population was community pharmacists registered with the Nepal Chemist and Druggist Association (NCDA) at Lalitpur district [24].

### Sampling method

The pharmacy shops were selected using systematic sampling with a sampling interval of three where the first pharmacy was selected randomly between one and three. The sampling interval was three as we were selecting 75 pharmacies from the 204 registered community pharmacies in the district.

### Sample size calculation

For sample size calculation, we assumed that the knowledge would be about 40% in our population of community pharmacists. This was obtained from the results of the pilot test and also from the literature review [25].

$$  {\text{Knowledge}} =40\%\,{\text{p}} = 0.4, {\text{q}} = 1-{\text{p}} = 0.6 $$

$$  {\text{N}}= [{\text{Z}}\alpha^{2}\,\times\,{\text{p}}\,\times\,{\text{q}}/({\text{M.E}}.)^{2}] $$

where Zα = 1.645 from normal table, two tailed at 10% alpha or 90% confidence interval, P = Population proportion, M.E = Margin of error = 10%, So, n = (1.645)^2^ × (0.4) × (0.6)/(0.1)^2^ = 65

Non response rate = 10%

Total sample size required after provision for drop outs from the study = 65 + 10% of 65 = 71

### Development of the questionnaire

The questionnaire included questions framed based on knowledge and attitude studies about pharmacovigilance and consumer pharmacovigilance conducted among community pharmacists in other countries. Manuscripts and published papers describing similar research and methodological issues were also studied [4, 27–30]. In the questionnaire, the issues addressed were the origin of pharmacovigilance in Nepal, progress and status of pharmacovigilance and the present system of pharmacovigilance in the country. Information about reporting ADRs, who can report ADRs, location and functions of the national pharmacovigilance center were also covered. Processing of ADRs and information about the scales used to analyze ADRs were also addressed in the questionnaire.

### Translation of the questionnaire

Additional file 1 shows the questionnaire developed in English. This questionnaire was translated into the Nepali language by two independent translators fluent in both languages and not associated with the study. The questionnaire was then back translated into English by two different individuals and the back translated version was compared with the original.

### Pretesting of the questionnaire

The questionnaire was tested for readability and ease of understanding among ten CPs (four rural and six urban). The data obtained was not included in the study. Face validation of the questionnaire was conducted by colleagues in the department. Then the questionnaire was sent to faculties of other departments for their inputs regarding readability and grammatical errors. Content validation was done by sending the questionnaire to content experts of pharmacovigilance within the country. Internal consistency was measured by calculating Cronbach’s alpha value, which was 0.67 indicating good reliability.

### Demographics

Gender, age, work experience, educational qualification and the location of the pharmacy were noted. Participant’s knowledge and attitude about pharmacovigilance and consumer pharmacovigilance was measured by noting his/her agreement with a set of 21 statements using a Likert-type scale.

### Information about the pharmacy

Information about the year of registration, number of patients visiting the pharmacy daily, number of books available for reference in the pharmacy, number of dispensers and total number of brands of medicines in the pharmacy were noted from the respondents.

### Scoring system

The scoring system used was: 5 = strongly agree with the statement, 4 = agree, 3 = neutral, 2 = disagree and 1 = strongly disagree with the statement using a Likert scale. There were twelve statements for assessing knowledge (maximum possible score of 60), and nine statements for attitude (maximum possible score of 45). The maximum possible overall score obtained by adding the ‘knowledge’, and ‘attitude’ scores was 105. The median and interquartile range were calculated for the ‘knowledge’, ‘attitude’ and “overall” scores.

### Conduct of the study

The questionnaire was administered personally by one of the investigator (RBP) visiting pharmacies in the study area. The community pharmacists were requested to complete the questionnaire in the presence of the investigator who could take back the completed questionnaire for analysis. This was used for collecting the baseline or pre intervention data. For the post intervention data, the questionnaire were administered to the participants immediately after the intervention session. Retention data were collected by visiting the participants individually in the same pharmacies 6 weeks after the educational intervention and employing the same procedure as that used for the pre-intervention data. Baseline knowledge and attitude of CPs was studied so that strengths and deficiencies could be noted and appropriate educational interventions planned. Knowledge and attitude was measured immediately after the educational intervention, and retention of information was tested 6 weeks post intervention.

### Educational intervention

The intervention included a session about pharmacovigilance and consumer pharmacovigilance. They were also asked to share their views and opinions about the adverse drug reaction reporting form designed for community pharmacists. Informative posters were also displayed for information sharing about ADRs. Participants also learned about the existing pharmacovigilance system in Nepal and the importance of involving consumers in the existing system. The duration of the session was 2 h. The teaching learning aids used were power point presentation, posters and leaflets about ADRs. The session was interactive and the participants designed an adverse drug reaction reporting form to be used by community pharmacists in the Nepali language.

### Statistical analysis

The knowledge, attitude and overall scores were tested for normality of distribution using Shapiro–Wilk test. The samples were noted not to follow a normal distribution so median and interquartile range and Wilcoxon Signed Rank Tests for Repeated Samples (non-parametric test) were used for comparing pre, post and retention scores. A p value less than 0.05 was taken as statistically significant. As the sample size was small KAP scores at the three points in time were compared only among a limited number of subgroups. The scores were compared among respondents grouped according to gender, location and qualifications as we felt these were the most important characteristics and there was enough number of respondents in each subgroup. The collected data was analyzed using SPSS version 20.0 for Windows.

### Ethical considerations

The study was approved by the Institutional Research Committee of KIST Medical College. All CPs were informed and explained about the aims and objectives of the study and invited to participate. Written informed consent was obtained from all participants.

## Results

Seventy-five of the 86 invited respondents participated and the response rate was 87%. Table 1 shows the respondent’s demographic characteristics. Male respondents were greater in number compared to females and respondents of age group 21–30 years were more than respondents from other age groups. Thirty-five respondents (46%) used Current Index of Medical Specialties (CIMS) as a reference book in the pharmacy while 13 respondents (17%) used Nepal drug review (NDR) as their reference. Twenty-five respondents (30%) had two people for dispensing medicines whereas a single person for dispensing was seen with 21 (28%) respondents. These people were alwayszfzfs present in the pharmacy.

**Table 1:** Demographic characteristics of respondents (n = 75)

Characteristic	Number (percentage)
Gender	
Male	45 (60)
Female	30 (40)
Age (in years)	
Below 20	12 (16)
21–30	39 (52)
>3	24 (32)
Work experience (year)	
<5	23 (30.6)
5–10	25 (33.4)
>10	27 (36)
Location	
Rural	40 (53.3)
Urban	35 (46.7)
Reference books available in the pharmacy	
CIMS	35 (46.6)
NDR	13 (17.4)
Both CIMS and NDR	16 (21.4)
None	11 (14.6)

Table 2 shows the knowledge, attitude and total (overall) scores at pre, post and retention stages with regard to the educational intervention for the participants. The knowledge score increased both immediately after and six weeks following the intervention. The overall score decreased 6 weeks post-intervention as attitude score had decreased significantly during the period.

**Table 2:** Differences in knowledge, attitude and total (overall) (shown as median (interquartile range) scores at pre, post and retention stages of the educational intervention

Domain	Pre	Post	p value	Pre	Retention	p value	Post	Retention	p value
Knowledge	39 (44–46)	44 (44–44)	<0.001	41 (40–41)	46 (43–46)	<0.001	46 (46–46)	53 (49–53)	0.254
Attitude	42 (40–44)	45 (44–46)	<0.001	42 (40–44)	35 (34–36)	<0.001	45 (44–46)	35 (34–36)	<0.001
Overall	81 (75–83)	89 (86–92)	<0.001	81 (75–83)	80 (77–83)	0.901	89 (86–92)	80 (77–83)	<0.001

Table 3 shows differences in overall, knowledge and attitude scores at pre, post and retention stages of the educational intervention according to gender and location of the community pharmacists as these variables had more than 30 samples in each category.

**Table 3:** Difference in scores among selected subgroups of community pharmacists before (pre-test), immediately after (post-test) and six weeks following (retention) an educational intervention

Characteristics	Pre-test median (interquartile range)	Post-test median (interquartile range)	p value^Δ^	Pre-test median (interquartile range)	Retention median (interquartile range)	p value^Δ^	Post-test median (interquartile range)	Retention median (interquartile range)	p value^Δ^
Overall score									
Gender									
Male	81 (75–83)	89 (85–92)	<0.0	81 (75–83)	80 (77–83)	1.000	89 (85–92)	80 (77–83)	<0.001
Female	80 (75–83)	91.5 (81–93)	<0.001	80 (75–83)	80 (75–82)	0.769	91.5 (81–93)	80 (75–82)	<0.001
Location									
Rural	80 (75–93)	90 (87–92.75)	<0.001	80 (75–93)	81 (79–93)	0.168	90 (87–92.75)	81 (79–93)	<0.001
Urban	82 (76–86)	89 (85–92)	<0.001	82 (76–86)	80 (75–93)	0.267	89 (85–92)	80 (75–93)	<0.001
Knowledge score									
Gender									
Male	39 (37–40)	44 (41–47)	<0.001	39 (37–40)	46 (42–48)	<0.001	44 (41–47)	46 (42–48)	0.097
Female	39 (37–42)	44 (43–45)	<0.001	39 (37–42)	45 (39–47)	<0.001	44 (43–45)	45 (39–47)	0.837
Location									
Rural	39 (44–46)	44 (44–44)	<0.001	39 (44–46)	46 (43–47)	<0.001	44 (44–44)	46 (43–47)	0.206
Urban	38 (33–46)	43 (43–44)	<0.002	38 (33–46)	46 (42–47)	<0.001	43 (43–44)	46 (42–47)	0.761
Attitude score									
Gender									
Male	43 (37–44)	44 (43–47)	0.012	43 (37–44)	35 (31–37)	0.003	44 (43–47)	35 (31–37)	<0.001
Female	41 (35–44)	46 (44–48)	<0.001	41 (35–44)	35 (31–37)	<0.001	46 (44–48)	35 (31–37)	<0.001
Location									
Rural	41 (35–44)	45 (43–47)	<0.001	41 (35–44)	35 (31–38)	<0.001	45 (43–47)	35 (31–38)	<0.001
Urban	43 (38–44)	45 (43–48)	0.039	43 (38–44)	34 (31–37)	<0.001	45 (43–48)	34 (31–37)	<0.001

^**Δ**^Wilcoxon Signed rank test

### Gender

The median knowledge scores improved immediately after the intervention and also six weeks following the educational intervention compared to the baseline among both genders. Males showed a consistent improvement in knowledge scores. Attitude scores improved after the intervention compared to baseline but the improvement was not significant 6 weeks after intervention among females. The overall scores also improved significantly after the intervention.

### Rural and urban location

Knowledge scores among community pharmacists working at rural and urban pharmacies improved significantly immediately after and 6 weeks post intervention. The attitude scores post intervention also improved but the improvement was higher among pharmacists working in rural areas. The overall scores improved significantly immediately post intervention.

## Discussion

The knowledge of community pharmacists in the present study regarding pharmacovigilance was low (47.5%). The topic of consumer pharmacovigilance was also new and not many were aware about it. This finding was similar to that observed in previous studies [26–31]. Community pharmacy practice is well developed in nations like the United Kingdom and Australia, where pharmacists are well aware of pharmacovigilance and consumer pharmacovigilance systems [32].

A possible reason for the low knowledge and attitude scores in the present study could be that not all persons working in community pharmacies were from the pharmacy profession. Some were nurses, community medicine auxiliaries (CMAs) while others were pharmacy professionals. CMAs are paramedical professionals who undergo basic medical training for eighteen months. They are capable of diagnosing and treating minor illnesses and can also refer the patients for higher specialized care services if needed and they do not have any legal authority to dispense medicines unless they have completed the DDA orientation training course [18]. In the existing pharmacovigilance system of Nepal, pharmacists are allowed to report ADRs in the hospital settings through the regional pharmacovigilance centres, but it has not yet being started for community settings. Inadequate knowledge of ADR reporting systems among community pharmacists has also been reported in the literature. In Saudi Arabia, 86.8% of community pharmacists surveyed were not aware of the country’s ADRs reporting program, and another study in Iran indicated that only 30% of pharmacists were aware about the Iranian pharmacovigilance centre [33–35].

Community pharmacists should be educated about pharmacovigilance in Nepal to improve their knowledge. Lack of knowledge about what is an adverse drug reaction, what is to be reported, who can and should report, when to report, how to report, where to report, whom to report and lack of availability of ADR reporting forms for community pharmacists as well as for consumers can hamper ADR reporting in community settings. The national pharmacovigilance program should use various interventional strategies followed by other countries for improving ADR reporting [36–40].

In our study, the attitude scores were good. In a study done by Khalili et al. [43], the attitude towards ADR reporting among community pharmacists improved after an intervention which has some similarities with studies done in the UK and other countries [11, 41–43], where pharmacists showed positive attitude towards ADR reporting. However, a study in Gujarat, India exploring KAP scores regarding pharmacovigilance among community pharmacists revealed a poor score and the authors had recommended an educational intervention [44].

The results of our study has shown that the educational program was effective in improving the knowledge and attitude of CPs towards pharmacovigilance. Similar findings were reported by previous studies [43, 45]. A statistical difference was noted between respondents’ gender and the responses to the statement adverse drug reactions are one of the major causes of death in the world and for the sentence about verbal reporting and written forms being preferred methods for ADR reporting (p < 0.001). Significant differences were also seen with the statement regarding involvement of Pharmaceutical industries in reporting ADRs and about the location of the national pharmacovigilance centre. Participant’s age, work experience, involvement in any kind of educational intervention has shown an impact on pharmacovigilance activities and ADR reporting as shown in other studies [30, 43, 45].

The baseline knowledge and attitude scores for both males and females were nearly same but the scores were better in females after the intervention. This was similar to that seen in a study done in Oman [46].

## Limitations

The study participants were from Lalitpur district and the result may not be generalizable to pharmacists other districts. Involving more pharmacist from the other two districts of the Kathmandu valley (Kathmandu and Bhaktapur) would have given a better understanding about the perception of community pharmacists from the valley. Also the overall sample was small and the intervention may not have been the only factor which changed attitudes, as no control group was used. Also some of the statements in the questionnaire were positive and may have influenced the participants’ response.

## Conclusion

The educational intervention improved the pharmacovigilance knowledge and attitude scores of the community pharmacists. The present study revealed an improvement in the knowledge scores for males and female participants whereas, the attitude scores improved among female respondents. Knowledge and attitude scores were higher among pharmacists working in rural areas.

Similar studies should be conducted among community pharmacists in other districts of Nepal. The perception of consumers regarding reporting ADRs to community pharmacists should also be studied.

## Additional file

**Additional file 1.** Questionnaire used in the study.

## Abbreviations

ADR: adverse drug reaction
CIMS: current index of medical specialties
HCP: Health care professionals
CP: community pharmacists
GDP: gross domestic product
DDA: Department of Drug Administration
NCDA: Nepal Chemist and Druggist Association
SPSS: statistical package for social sciences
NDR: Nepal drug review
NGO: Non-governmental organization
CMA: community medicine auxiliary
B.Pharm: Bachelor in pharmacy
M.Pharm: Masters in pharmacy
D.Pharm: Diploma in pharmacy
