# Supplementary material for: Effect of an educational intervention on knowledge and attitude regarding pharmacovigilance and consumer pharmacovigilance among community pharmacists in Lalitpur district, Nepal
Source: BMC Res Notes. 2017 Jan 3;10:4. doi: 10.1186/s13104-016-2343-5 (PMC5217211; doi:10.1186/s13104-016-2343-5)
Supplement: Supplementary file 1 — Additional file 1. Questionnaire used in the study. [file 13104_2016_2343_MOESM1_ESM.docx]

__________________________________________________________________

**The knowledge, and attitude (KA) of community pharmacists in Lalitpur district, Nepal about consumer pharmacovigilance and adverse drug reaction reporting**

______________________________________________________________________

Details of the responder and the pharmacy

Name: Age: Gender: Male/Female

Professional qualification : Pharmacy Name: Date:

Length of experience: Number of pharmacists working:

Average No of patients per day: Year of starting the Pharmacy:

No of persons involved in dispensing: Location of the Pharmacy:

Available source/s for information about medicines: Total number of preparations: _____________________________________________________________

***For the following statements score using the following key (1 = strongly disagree with the statement, 2= disagree with the statement, 3= neutral, 4= agree with the statement, 5= strongly agree with the statement.) Use whole numbers only.***

1. A drug may take more than 12 months to reach the market. - K
2. Medicines can cause beneficial as well as harmful effects. -K
3. Adverse drug reactions can even cause death in humans. - K
4. Department of Drug Administration (DDA) is the organization in Nepal involved in drug safety issues.- K
5. Consumer pharmacovigilance is already established in Nepal.-K
6. The Government in Nepal should play an important role in the success of consumer pharmacovigilance. -A
7. Patients can themselves report ADR to doctors and other health care professionals. -A
8. Herbal medicines are as likely to cause adverse drug reactions as allopathic medicines. -A
9. ADR reporting can cause loss of my useful time. -A
10. The Department of drug administration should take steps for facilitating reporting of ADRs by consumers in Nepal. -A
11. Pharmaceutical Industries should also report ADRs. -A
12. Paracetamol can also cause adverse drug reactions.-K
13. The national center for reporting adverse drug reactions is Department of Drug Administration, located at Babarmahal, Kathmandu. - K
14. Adverse drug reactions can be of different types. -A
15. Headache, diarrhea, allergy can be regarded as examples of adverse drug reactions.-K
16. Reading articles on adverse drug reactions will be beneficial to me as a community pharmacist. -A
17. Consumers are not aware enough about adverse reactions to the medicines taken by them.-K
18. Consumer reporting will increase the knowledge of the consumer about ADRs.-A
19. Consumer reporting will promote consumer rights for health in Nepal.-A
20. Media and journalists can play an important role in the success of consumer reporting in Nepal.-K
21. Non-governmental organizations (NGOs) in Nepal can help and play an important role in the success of a consumer reporting programme.-K

Any comments:………………………………………………………………………………………………

…………………………………………………………………………………………………………………………………………………………………………………………………………………………

K – statement measuring knowledge, A- statement measuring attitude
